# The Alteration of Circulating Lymphocyte Subsets During Tacrolimus Therapy in Neuromyelitis Optica Spectrum Disorder and Its Correlation With Clinical Outcomes

**DOI:** 10.3389/fneur.2021.816721

**Published:** 2022-01-19

**Authors:** Liang Wang, Wenjuan Huang, Jingzi ZhangBao, Xuechun Chang, Hongmei Tan, Lei Zhou, Chuanzhen Lu, Min Wang, Jiahong Lu, Chongbo Zhao, Chao Quan

**Affiliations:** ^1^Department of Neurology, Huashan Hospital, Fudan University, Shanghai, China; ^2^National Center for Neurological Disorders (NCND), Shanghai, China; ^3^Department of Ophthalmology and Vision Science, Eye and ENT Hospital, Fudan University, Shanghai, China

**Keywords:** tacrolimus, neuromyelitis optica spectrum disorder, lymphocyte subset, clinical outcome, correlation

## Abstract

**Objectives:**

We aimed to explore the alteration of circulating lymphocyte subsets before and after tacrolimus (TAC) therapy in neuromyelitis optica spectrum disorder (NMOSD) and its correlation with clinical outcomes.

**Methods:**

Anti-aquaporin-4 antibody (AQP4-ab)-positive patients with NMOSD treated with TAC were followed and clinically evaluated at 0, 3, 6, and 12 months after initiation of TAC. Flow cytometry was employed to detect the proportion of various whole blood lymphocyte subsets at every time point. Correlation analysis was further performed to explore the association between annualized relapse rate (ARR), the Expanded Disability Status Scale (EDSS) score, and the proportion of circulating lymphocyte subsets before and after TAC therapy.

**Results:**

A total of 13 eligible patients with NMOSD were included. The proportion of CD19^+^CD24^hi^CD38^hi^/CD19^+^ and CD19^+^CD5^+^CD1d^hi^/CD19^+^ lymphocyte subsets increased significantly after TAC therapy (*p* = 0.010 and *p* < 0.001). The proportion of CD19^+^BAFFR^+^, CD19^+^IFN-γ^+^, and CD19^+^IL-10^+^ subsets decreased significantly after TAC therapy (*p* = 0.015, 0.018, and 0.042, respectively). There was a negative correlation between CD4^+^CD25^hi^ subset and EDSS score (*p* = 0.016, *r* = −0.652).

**Conclusion:**

Possibly through increasing regulatory B and suppressing BAFFR^+^ B and interferon (IFN)-γ^+^ B subsets, TAC could decrease relapse. EDSS score may be correlated with some lymphocyte subsets after TAC therapy.

## Introduction

Neuromyelitis optica spectrum disease (NMOSD) is one of the central nervous systems inflammatory diseases mainly mediated by antiaquaporin-4 antibody (AQP4-ab) and involving the optic nerve, spinal cord, and specific brain regions (1). Previous immunological studies have shown that NMOSD was dominated by humoral immunity, with AQP4-ab deposition and complement activation in the lesions (2, 3).

Numerous B cell subsets play important roles in the pathogenesis of NMOSD, including naïve B cells, regulatory B cells, memory B cells, and plasmablasts (4). Follicular helper T cells (Tfh) could promote the differentiation of B cells in the germinal center into memory B cells and plasma cells, thereby participating in the pathogenesis of NMOSD (5). It was generally believed that T helper cell 17 (Th17)-related cytokines such as interleukin-17 (IL-17) and B cell cytokines such as B-cell-activating factor (BAFF) were elevated in the serum and cerebrospinal fluid of patients with NMOSD, and whether there were abnormalities in the number and function of regulatory T cells in NMOSD was still controversial (6, 7). Studies have found that the proportion of Tfh in NMOSD during relapse was significantly higher than that in the remission period and healthy controls while the proportion of regulatory B cells and IL-10 during the acute attack was significantly lower than healthy controls (8, 9). High-dose methylprednisolone therapy in NMOSD could significantly reduce the proportion of Tfh and interferon-γ (IFN-γ) in B cells (8, 10).

Tacrolimus (TAC) was originally a macrolide compound extracted from the genus Streptomyces tsukubanesis. Although TAC and cyclosporin A (CsA) both belong to calcineurin inhibitors, the potency of TAC is 10–100 times of CsA (11). TAC plays a major role in cellular immunity. It binds to TAC binding protein 12 in the cell and forms a complex to inhibit phosphatase activity, thereby preventing the dephosphorylation and translocation of nuclear factor of activated T cell, inhibiting the transcription of cytokines including IL-2, interfering with the differentiation and proliferation of T cells, thereby inhibiting the inflammation and alleviating the symptoms of autoimmune diseases (12). Studies have shown that TAC could specifically inhibit the number and proportion of Tfh in lymph nodes and blood in kidney transplant patients without affecting regulatory T cells and other subgroups (13). In addition, the proportion of CD19^+^BAFFR^+^ cells in patients with myasthenia gravis (MG) decreased after taking TAC (14).

Tacrolimus was previously applied in the field of solid organ transplantation (15). There were also studies using TAC as a maintenance therapy for NMOSD in remission, which proved to be effective in preventing the relapse of NMOSD, reducing annual relapse rate (ARR) and the Expanded Disability Status Scale (EDSS) score (16–18). Tacrolimus was less likely to cause leukopenia and liver function damage, making it one of the alternatives for patients who were intolerant of azathioprine (AZA) or mycophenolate mofetil (MMF). Although the imbalance of Tfh and regulatory B cells has been clarified in the immunopathogenesis of NMOSD, there still lacked whether the effectiveness of TAC was related to it. In this study, we aimed to explore the correlation between clinical outcomes and immunological measures during TAC therapy.

## Materials and Methods

### Participants and Data Collection

This was a single-center prospective observational cohort study. Patients with NMOSD taking TAC (3 mg/d) as maintenance therapy were prospectively included in the Department of Neurology of Huashan Hospital from December 2017 to October 2018. The inclusion criteria were: (1) taking TAC (3 mg/d) combined with or without small dose oral glucocorticoid; (2) not simultaneously receiving other immunosuppressive treatment such as AZA, MMF, cyclophosphamide (CTX), and rituximab (RTX); (3) meeting the diagnostic criteria of NMOSD established by the International Panel in 2015; (4) age ≥ 18 years. Demographics and clinical data were collected, including the number of patients, gender, onset age, disease duration, disease course, serum AQP4-ab or MOG-ab titer, and previous immunotherapy. These patients were followed-up and evaluated during the baseline, 3, 6, and 12 months after TAC initiation to record relevant outcomes, including ARR and EDSS scores before and after treatment. Blood samples were taken to detect the proportion of whole blood lymphocyte subsets during each follow-up. We confirmed that no symptoms or data related to infection were detected when blood samples were obtained. During TAC therapy, if the patient had relapsed and received high-dose methylprednisolone therapy, it would affect the proportion of lymphocyte subsets, which were not included in further analysis. Those discontinuing TAC or changing the immunosuppressive agent because of the adverse events within 1 year would not be included in further analysis.

### AQP4-ab and MOG-ab Detection

All patients at Huashan Hospital had undergone serum AQP4-ab and MOG-ab detection using fixed cell-based indirect immune-fluorescence test (Euroimmun AG, Lüebeck, Germany) as part of a routine diagnostic approach.

### Flow Cytometry Analysis

We separated 200 μl of EDTA-anticoagulated whole venous blood into four tubes. Each was immunostained with fluorescent-labeled monoclonal antibodies for 30 min at 4°C in darkness. After red blood cell lysis using FACS Lysing Solution (BD Biosciences, San Jose, California, USA), the samples were washed twice and resuspended in 200 μl of phosphate-buffered saline (PBS) supplemented with 0.5% fetal bovine serum (FBS). The frequencies of different lymphocyte subsets were determined with Attune Acoustic Focusing Cytometer (Thermo Fisher Scientific, Waltham, MA, USA). Isotype controls were performed to establish appropriate gating (Table 1).

**Table 1:** Detection of lymphocyte subsets by flow cytometry.

**Lymphocyte subsets**	**Combinations of monoclonal**
	**antibody**
CD19^+^CD27^+^ memory B cell	CD19 PE-Cy7, CD27 FITC
CD19^+^CD27^−^ naïve B cell	CD19 PE-Cy7, CD27 FITC
CD19^+^CD24^hi^CD38^hi^ regulatory B cell	CD19 PE-Cy7, CD24 PE, CD38 APC
CD19^+^CD5^+^CD1d^hi^ regulatory B cell	CD19 PE-Cy7, CD5 APC, CD1d PE
CD4^+^CXCR5^+^ICOS^+^ follicular helper T cell	CD4 FITC, CXCR5 PE-Cy7, ICOS APC
CD4^+^CD25^hi^ regulatory T cell	CD4 FITC, CD25 PE
CD19^+^BAFFR^+^ B cell	CD19 PE-Cy7, BAFFR PE
CD4^+^IL-17^+^ T cell	CD4 FITC, IL-17 PE
CD19^+^IFN-γ^+^ B cell	CD19 PE-Cy7, IFN-γ FITC
CD19^+^IL-10^+^ B cell	CD19 PE-Cy7, IL-10 PE

### Cytokine Detection

Ficoll-Hypaque density gradient centrifugation (Sigma Aldrich, St. Louis Missouri, USA) was performed to separate peripheral blood mononuclear cells (PBMCs) from 6 ml of heparinized blood. Cells from the interface were collected and washed with PBS. Then the cell concentration was adjusted to 1 × 10^6^ cells/ml in RPMI 1,640 including L-glutamine and NaHCO_3_ (Sigma Aldrich, St. Louis, Missouri, USA) supplemented with 100 U/ml penicillin, 0.1 mg/ml streptomycin (Life Technologies, Carlsbad, California, USA) and 10% FBS in 24-well U-bottom plates (Nunc, Langenselbold, Germany). To increase surface expression of B cells, the PBMCs were stimulated with 0.1 mg/ml CpG oligodeoxynucleotide (CpG ODN) 2006 (InvivoGen, San Diego, California, USA) at 37°C with 5% CO_2_ for 22 h. During the last 5 h, a 2 μl/ml cell stimulation cocktail (eBioscience, San Jose, California, USA) was added. Then, the cells were immunostained with antihuman CD4 FITC or CD19 PE-Cy7 (eBioscience, San Jose, California, USA) for T or B cells, respectively. Subsequently, the cells were washed, fixed, and permeabilized with the Fix&Perm Kit (Invitrogen, Camarillo California, USA). Antihuman IL-17-PE or IFN-γ FITC and IL-10 PE (eBioscience, San Jose California, USA) were added to the permeabilized T or B cells for intracellular cytokine detection. Appropriate isotype controls were conducted to determine the cytokine detection gates.

### Statistical Analysis

In this study, FlowJo X10.0 (FlowJo, LLC., Ashland, Oregon) was used to gate and analyze the FCS files. The data were analyzed with SPSS 22.0 software (SPSS Inc., Chicago IL, USA) while figures were made with Graphpad Prism 6 software (GraphPad Software inc., La Jolla CA, USA). Counting data was expressed as count (%). Quantitative data conforming to normal distribution were expressed as the mean ± SD, and one-way repeated measurement ANOVA was applied to compare the proportion of lymphocytes during each follow-up. Bonferroni method was used to correct the significance level of *posthoc* pairwise comparison. Non-normally distributed quantitative data were presented as median and range, and Friedman rank-sum test was applied to compare the proportion of lymphocytes during each follow-up. Spearman or Pearson correlation analysis was used for the correlation between clinical outcomes and flow cytometry assessment. Correlation and clustering analysis was performed with the corr package in R, version 4.0.3 (http://www.r-project.org/). Statistical significance was set at *p* < 0.05.

## Results

### Demographic and Clinical Findings

There were 20 patients with NMOSD taking TAC prospectively. After excluding five patients having relapse within 1 year and 2 patients with MOG-ab or seronegative status, 13 AQP4-ab-positive patients were included in this study, with antibody titer ranging from 1:10 to 1:1,000. There were 11 patients taking oral glucocorticoid as a combined therapy. The average onset age was 40.6 ± 15.8 years while the disease course was 42.9 ± 20.6 months. A total of nine patients had a relapsing course while the other patients had a monophasic course before TAC therapy. Five patients used to take immunosuppressive agents and experienced 4–8 weeks of washout period. During the 12 months follow-up period after TAC initiation, no patients experienced NMOSD relapse. The EDSS score and ARR before TAC therapy were 4 (1–5.5) and 1 (0–3). After 12 months of TAC therapy, the EDSS score and ARR were 3 (0–4) and 0 (0–0). The demographical and clinical characteristics of this cohort were listed in Table 2.

**Table 2:** Demographic and clinical characteristics of patients with NMOSD taking TAC.

**No. of**	**Sex**	**Onset**	**Disease**	**Disease**	**Antibody titer**	**EDSS score**	**ARR before-**	**Previous**	**Combined therapy**
**patient**		**age (y)**	**duration (m)**	**course**		**before-after therapy**	**after therapy**	**therapy**	**at last follow-up**
1	F	48.7	29.0	Relapse	AQP4-ab 1:32	4-4	3-0	None	Prednisone 1#qd
2	F	62.0	49.0	Relapse	AQP4-ab 1:100	5.5-4	1-0	None	Prednisone 5#qd
3	F	46.0	21.9	Monophase	AQP4-ab 1:100	4-3	1-0	None	None
4	F	41.7	90.0	Relapse	AQP4-ab 1:10	1-0	2-0	AZA	Prednisone 2#qd
5	F	57.6	19.0	Monophase	AQP4-ab 1:32	4-3	1-0	None	None
6	F	19.4	64.5	Monophase	AQP4-ab 1:1000	4-4	0-0	CTX	None
7	F	34.2	50.0	Relapse	AQP4-ab 1:100	1-0	1-0	None	None
8	F	28.4	20.0	Relapse	AQP4-ab 1:320	4-4	2-0	None	None
9	F	67.9	22.0	Monophase	AQP4-ab 1:32	5-1.5	1-0	AZA	None
10	F	25.3	42.0	Relapse	AQP4-ab 1:10	4-4	2-0	AZA	None
11	F	46.4	53.0	Relapse	AQP4-ab 1:32	3-2	1-0	None	None
12	F	25.8	46.0	Relapse	AQP4-ab 1:32	1-0	1-0	AZA	None
13	F	24.1	51.0	Relapse	AQP4-ab 1:32	2-1	1-0	None	None

*NMOSD, neuromyelitis optica spectrum disorder; TAC, tacrolimus; AQP4-ab, anti-aquaporin 4 antibody; AZA, azathioprine; CTX, cyclophosphamide; y, year; m, month; EDSS, Expanded Disability Status Scale; ARR, annualized relapse rate*.

### Memory, Naïve, Regulatory, and BAFFR^+^B Cells

**Figure 1:**
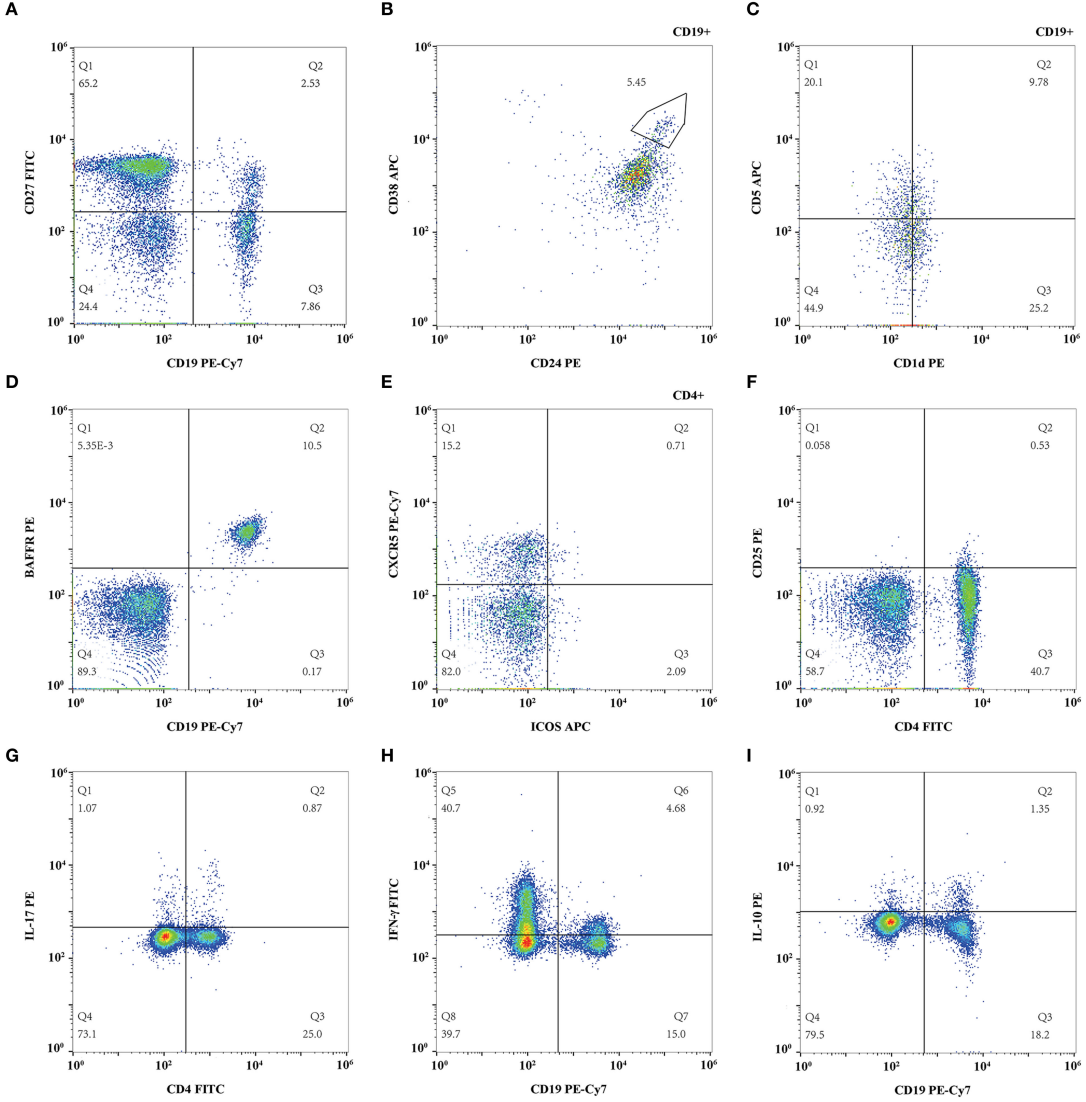
Representative flow cytometry figure of lymphocyte subsets. **(A)** CD19^+^CD27^+^ memory B cell and CD19^+^CD27^−^ naïve B cell; **(B)** CD19^+^CD24^hi^CD38^hi^ regulatory B cell; **(C)** CD19^+^CD5^+^CD1d^hi^ regulatory B cell; **(D)** CD19^+^BAFFR^+^ B cell; **(E)** CD4^+^CXCR5^+^ICOS^+^ follicular helper T cell. **(F)** CD4^+^CD25^hi^ regulatory T cell; **(G)** IL-17 expressing CD4^+^ T cell; **(H)** Interferon (IFN)-γ expressing CD19^+^ B cell; **(I)** IL-10 expressing CD19^+^ B cell.

**Figure 2:**
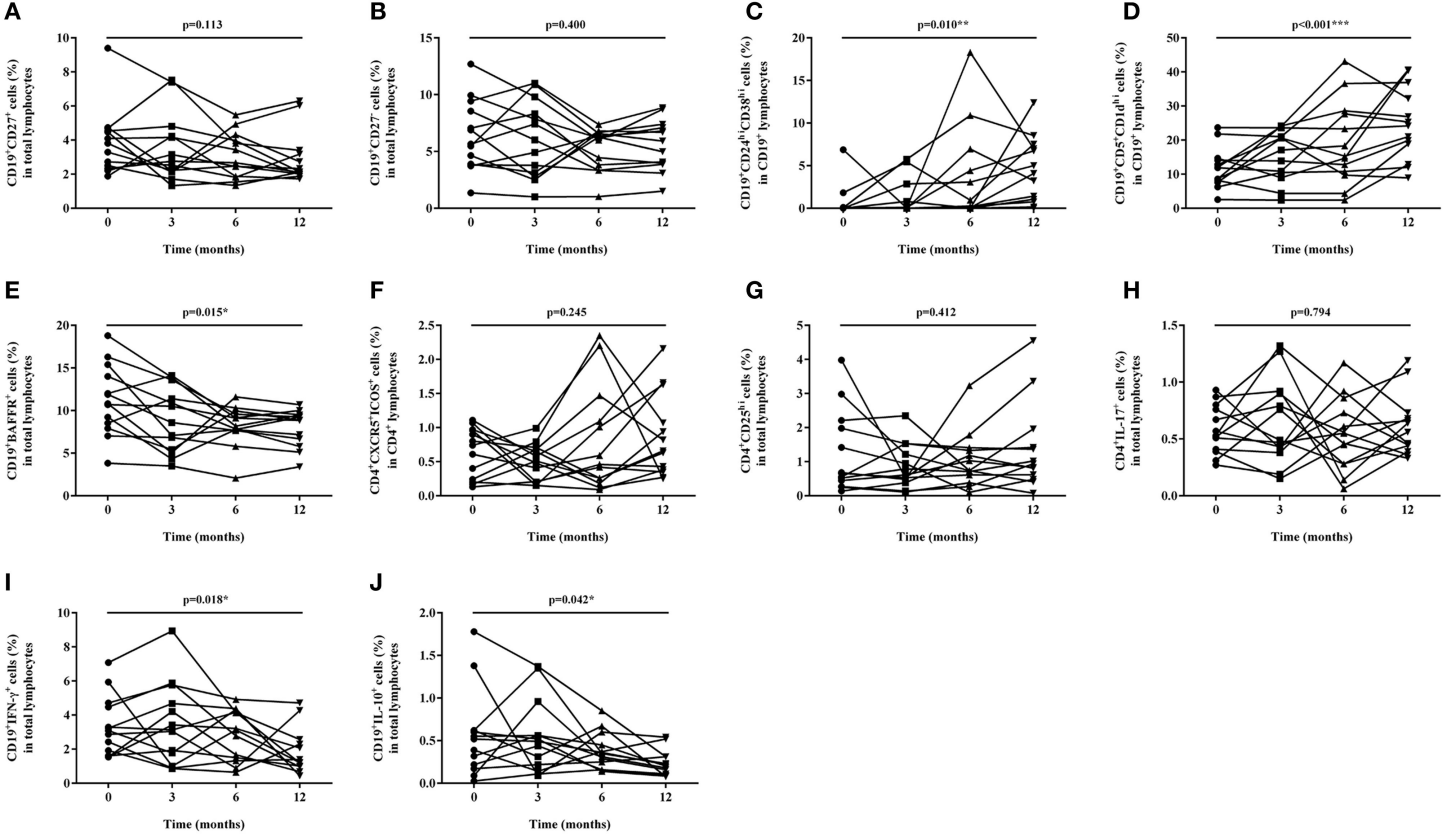
Proportion changes of lymphocyte subsets during baseline and after 3, 6, and 12 months of tacrolimus (TAC) therapy. **(A)** CD19^+^CD27^+^ memory B cell; **(B)** CD19^+^CD27^−^ naïve B cell; **(C)** CD19^+^CD24^hi^CD38^hi^ regulatory B cell; **(D)** CD19^+^CD5^+^CD1d^hi^ regulatory B cell; **(E)** CD19^+^BAFFR^+^ B cell; **(F)** CD4^+^CXCR5^+^ICOS^+^ follicular helper T cell. **(G)** CD4^+^CD25^hi^ regulatory T cell; **(H)** IL-17 expressing CD4^+^ T cell; **(I)** IFN-γ expressing CD19^+^ B cell; **(J)** IL-10 expressing CD19^+^ B cell.

The proportions of CD19^+^CD27^+^ memory B cells, CD19^+^CD27^−^ naïve B cells, CD19^+^CD24^hi^CD38^hi^, and CD19^+^CD5^+^CD1d^hi^ regulatory B cells were measured and compared during each follow-up. The proportion of CD19^+^CD27^+^ memory B cells in total lymphocytes decreased from 3.90 ± 1.95% to 2.92 ± 1.52% gradually (*p* = 0.113) (Figures 1A, 2A) while the percentage of CD19^+^CD27^−^ naïve B cells in total lymphocytes reduced from 6.39 ± 3.12% to 5.61 ± 2.23% (*p* = 0.400), which both did not change significantly (Figures 1A, 2B) after TAC therapy. The frequencies of CD19^+^CD24^hi^CD38^hi^ regulatory B cells in CD19+ B lymphocytes increased from 0.69 ± 1.92% to 4.45 ± 3.83% (*p* = 0.010) (Figures 1B, 2C), while CD19^+^CD5^+^CD1d^hi^ regulatory B cells in CD19^+^ B lymphocytes enhanced from 11.76 ± 5.94% to 24.63 ± 10.52% with highly statistical significance after TAC therapy (*p* < 0.001) (Figures 1C, 2D). Compared with baseline, *posthoc* pairwise comparison exhibited the differences of CD19^+^CD24^hi^CD38^hi^ regulatory B cells (*p* = 0.023) and CD19^+^CD5^+^CD1d^hi^ regulatory B cells (*p* = 0.002) were both statistically significant in 12 months. The proportion of CD19^+^BAFFR^+^ cells in total lymphocytes had a gradual decrease from 11.26 ± 4.13% to 8.00 ± 2.18% with statistical significance after TAC therapy (*p* = 0.015) (Figures 1D, 2E). And the difference nearly reached statistical significance in 12 months compared to baseline in *posthoc* pairwise comparison (*p* = 0.057).

### Tfh and Regulatory T Cells

The proportions of CD4^+^CXCR5^+^ICOS^+^ Tfh and CD4^+^CD25^hi^ regulatory T cells were measured and compared during each follow-up. The percentage of CD4^+^CXCR5^+^ICOS^+^ Tfh in CD4^+^ T lymphocytes enhanced from 0.64 ± 0.37% to 0.87 ± 0.61% (*p* = 0.412) (Figures 1E, 2F), while CD4^+^CD25^hi^ regulatory T cells in total lymphocytes increased from 1.24 ± 1.21% to 1.37 ± 1.27% (*p* = 0.412) (Figures 1F, 2G), which both did not change significantly after TAC therapy.

### The Expression of Cytokines in Circulating T and B Cells

The proportion of IL-17 expressing T cells in the total lymphocytes changed slightly from 0.58 ± 0.21% to 0.61 ± 0.27%, which exhibited no statistical difference after TAC therapy (*p* = 0.794) (Figures 1G, 2H). The frequencies of circulating IFN-γ expressing B cells in the total lymphocytes decreased from 3.36 ± 1.74% to 1.79 ± 1.37% significantly (*p* = 0.018) (Figures 1H, 2I) while IL-10 expressing B cells in the total lymphocytes decreased from 0.56 ± 0.50% to 0.23 ± 0.15 % significantly (*p* = 0.042) (Figures 1I, 2J) after TAC therapy, although, in a *posthoc* analysis, the difference did not reach statistical significance in 12 months compared with baseline.

### Correlation and Clustering Analysis

**Figure 3:**
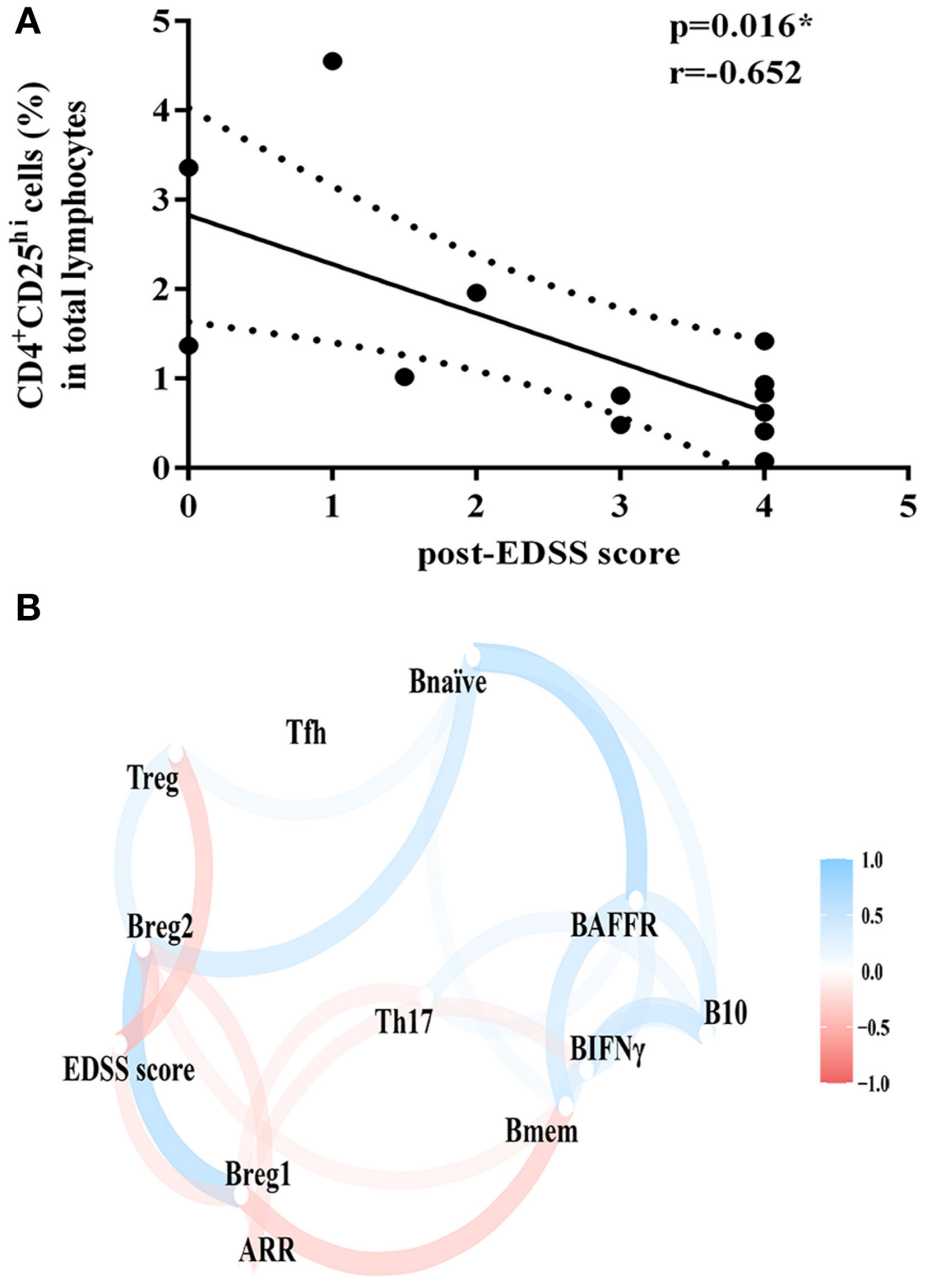
Correlation analysis of clinical outcomes and proportion of lymphocyte subsets. **(A)** Correlation of EDSS score after TAC therapy and the proportion of CD4^+^CD25^*hi*^ cell subsets. **(B)** Correlation and clustering analysis of all lymphocyte subsets and clinical outcomes during TAC therapy. Bmem, CD19^+^CD27^+^ memory B cell; Bnaïve, CD19^+^CD27^+^naïve B cell; Breg1, CD19^+^CD24^*hi*^ CD38^*hi*^ regulatory B cell; Breg2, CD19^+^CD5^+^CD1d^*hi*^ regulatory B cell; BAFFR, CD19^+^BAFFR^+^ B cell; Tfh, CD4^+^CXCR5^+^ICOS^+^ follicular helper T cell; Treg, CD4^+^CD25^*hi*^ regulatory T cell; Th17, IL-17 expressing CD4^+^ T cell; BIFNγ, IFN-γ expressing CD19^+^ B cell; B10, IL-10 expressing CD19^+^ B cell; EDSS, Expanded Disability Status Scale; ARR, annualized relapse rate. Variables that were more highly correlated appeared closer together and were connected by stronger paths. Paths were also colored by their sign (blue for positive and red for negative). The proximity of the points was determined through multidimensional clustering.

We found that the EDSS score after TAC therapy was negatively correlated with the proportion of CD4^+^CD25^hi^ cell subsets (*p* = 0.016, *r* = −0.652) (Figure 3A). We did not observe the correlation between the change in EDSS score or ARR and the change in the ratio of lymphocyte subsets after TAC therapy. The correlation and clustering analysis of all lymphocyte subsets and clinical outcomes during TAC therapy were exhibited in Figure 3B.

## Discussion

This study preliminarily explored the influence of TAC on the proportion of circulating lymphocytes. TAC may reduce relapse by increasing the proportion of regulatory B cells and inhibiting the proportion of BAFFR^+^B cells and IFN-γ^+^ B cells. EDSS score may be correlated with some lymphocyte subsets before and after TAC therapy.

Memory B cells were generally classified as CD19^+^CD27^+^, which mainly secreted inflammatory cytokines such as IFN-γ and tumor necrosis factor α (TNF-α) while seldom secreted anti-inflammatory cytokines such as IL-10, thereby promoting the relapse of NMOSD. And naïve B cells were mainly defined as CD19^+^CD27^−^, which mainly secreted IL-10 while seldom secreted IFN-γ, thereby inhibiting the relapse of NMOSD (4, 19). Compared with the aforementioned studies, our research results further showed that the proportion of circulating memory B cells gradually decreased during TAC therapy. Although the difference was not statistically significant (*p* = 0.113) possibly from the small sample size, this result could explain the decreased secretion of IFN-γ with a highly statistically significant difference (*p* = 0.018). The proportion of circulating naïve B cells had a decreased trend during TAC therapy, and the difference did not reach statistical significance (*p* = 400). As the proportion of B cells secreting IFN-γ decreased, this could explain the similar decrease in the proportion of B cells secreting IL-10, and the difference reached statistical significance (*p* = 0.042). Previous studies demonstrated that the serum IFN-γ and IL-10 in patients with MG under TAC therapy were reduced, which was consistent with our study (14). Therefore, suppressing the proportion of memory B cells may be one of the mechanisms by which TAC exerted an immunosuppressive effect.

Regulatory B cells had different subsets including CD19^+^CD24^hi^CD38^hi^ and CD19^+^CD5^+^CD1d^hi^, with the function of secreting IL-10, inducing the function of regulatory T cells, inhibiting Th1, Th17, effector T cells, monocytes, and dendritic cells (20). Studies have shown that the ratio of CD19^+^CD24^hi^CD38^hi^ and IL-10 levels during the acute attack stage were significantly lower than those of healthy control (9). Compared with the aforementioned results, ours further demonstrated that the ratio of CD19^+^CD24^hi^CD38^hi^ and CD19^+^CD5^+^CD1d^hi^ increased during TAC therapy with statistical significance (*p* = 0.010 and *p* < 0.001, respectively). Therefore, increasing the proportion of regulatory B cells may be another mechanism by which TAC performed an immunosuppressive function.

BAFF maintained the survival and differentiation of B cells by binding BAFFR which was mainly expressed on the surface of B cells (21). Previous studies have shown that the levels of cerebrospinal fluid and serum BAFF in patients with NMOSD were increased, which was positively correlated with the EDSS score (22). Studies have also shown that the proportion of CD19^+^BAFFR^+^ cells in patients with MG increased, while the proportion of CD19^+^BAFFR^+^ cells in patients with MG decreased after taking TAC, which was correlated with the improvement of clinical symptoms (14, 23). Similar to the aforementioned results, our study found that the proportion of CD19^+^BAFFR^+^ cells decreased during TAC therapy, and the difference was statistically significant (*p* = 0.015). But we did not find a correlation between CD19^+^BAFFR^+^ cells and ARR or EDSS score. This may come from the different pathogenesis and outcome measures of MG and NMOSD.

Tfh, which could help B cells differentiate into memory B cells and plasma cells, played a very important role in autoimmune diseases. Tfh had many types and CD4^+^CXCR5^+^ICOS^+^ was one of the subsets (24). Studies have found that the proportion of follicular helper T cells during relapse was significantly higher than that in the remission period and healthy control (8). It was generally believed that Th17 cell-related cytokines and chemokines in serum and cerebrospinal fluid of NMOSD were elevated, and whether there were abnormalities in the number and function of regulatory T cells in NMOSD was still controversial (6, 7). Previous studies have found that TAC could specifically inhibit the number and proportion of Tfh in lymph nodes and blood in kidney transplant patients without affecting regulatory T cells and other subpopulations (13). Similar to the aforementioned results, we did not find differences in the ratio of CD4^+^CD25^hi^ cells, CD4^+^CXCR5^+^ICOS^+^ cells, and CD4^+^IL-17^+^ cells during TAC therapy (*p* = 0.412, 0.245, and 0.794, respectively). The reason might be that the patient was in the remission period and the proportion of T cells in the aforementioned three groups was relatively small. However, we found that the EDSS score after treatment had a negative correlation with the proportion of CD4^+^CD25^hi^ cells (*p* = 0.016, *r* = −0.652). Therefore, the changes in the proportion of T cells in the aforementioned three groups might affect the clinical outcomes of NMOSD after TAC therapy.

Our study had some limitations. First, the number of included patients and follow-up time were limited, therefore, the immunological measures before and after TAC therapy could not be fully evaluated. Second, some patients took small doses of prednisone during TAC therapy, which may have an impact on the immunological measures. In addition, some patients having relapses during TAC therapy were not included. Studies with a larger sample size were needed to further analyze the correlation between clinical and immunological measures.

## Conclusion

Possibly through increasing regulatory B and suppressing BAFFR^+^ B and IFN-γ^+^ B subsets, TAC could decrease relapse. EDSS score may be correlated with some lymphocyte subsets after TAC therapy.

## Data Availability Statement

The raw data supporting the conclusions of this article will be made available by the authors, without undue reservation.

## Ethics Statement

The studies involving human participants were reviewed and approved by Medical Ethics Committee of Huashan Hospital within the Shanghai Medical College at Fudan University. The patients/participants provided their written informed consent to participate in this study.

## Author Contributions

LW designed and conceptualized the study, interpreted and analyzed the data, and drafted and revised the manuscript for intellectual content. WH, JZ, XC, HT, and LZ played a major role in the acquisition of data and revised the manuscript for intellectual content. CL, MW, JL, and CZ revised the manuscript for intellectual content. CQ designed and conceptualized the study, interpreted and analyzed the data, and revised the manuscript for intellectual content. CQ had full access to all the data in the study and had final responsibility for the decision to submit for publication. All authors contributed to the article and approved the submitted version.

## Funding

This research was supported by the National Natural Science Foundation of China (Grant Nos. 82171341 and 81771296), the Shanghai Municipal Science and Technology Major Project (No. 2018SHZDZX01) and ZHANGJIANG LAB, and the National Key Research and Development Program of China (2016YFC0901504).

## Conflict of Interest

The authors declare that the research was conducted in the absence of any commercial or financial relationships that could be construed as a potential conflict of interest.

## Publisher's Note

All claims expressed in this article are solely those of the authors and do not necessarily represent those of their affiliated organizations, or those of the publisher, the editors and the reviewers. Any product that may be evaluated in this article, or claim that may be made by its manufacturer, is not guaranteed or endorsed by the publisher.
